# Quantifying impairment and disease severity using AI models trained on healthy subjects

**DOI:** 10.1038/s41746-024-01173-x

**Published:** 2024-07-06

**Authors:** Boyang Yu, Aakash Kaku, Kangning Liu, Avinash Parnandi, Emily Fokas, Anita Venkatesan, Natasha Pandit, Rajesh Ranganath, Heidi Schambra, Carlos Fernandez-Granda

**Affiliations:** 1 Center for Data Science, New York University, 60 Fifth Ave, New York, NY 10011 USA; 2 Department of Neurology, NYU Grossman School of Medicine, 550 1st Ave, New York, NY 10016 USA; 3 Department of Rehabilitation Medicine, NYU Grossman School of Medicine, 550 1st Ave, New York, NY 10016 USA; 4 Courant Institute of Mathematical Sciences, New York University, 251 Mercer St, New York, NY 10012 USA

**Keywords:** Neurological disorders, Predictive markers, Osteoarthritis

## Abstract

Automatic assessment of impairment and disease severity is a key challenge in data-driven medicine. We propose a framework to address this challenge, which leverages AI models trained exclusively on healthy individuals. The COnfidence-Based chaRacterization of Anomalies (COBRA) score exploits the decrease in confidence of these models when presented with impaired or diseased patients to quantify their deviation from the healthy population. We applied the COBRA score to address a key limitation of current clinical evaluation of upper-body impairment in stroke patients. The gold-standard Fugl-Meyer Assessment (FMA) requires in-person administration by a trained assessor for 30-45 minutes, which restricts monitoring frequency and precludes physicians from adapting rehabilitation protocols to the progress of each patient. The COBRA score, computed automatically in under one minute, is shown to be strongly correlated with the FMA on an independent test cohort for two different data modalities: wearable sensors (*ρ* = 0.814, 95% CI [0.700,0.888]) and video (*ρ* = 0.736, 95% C.I [0.584, 0.838]). To demonstrate the generalizability of the approach to other conditions, the COBRA score was also applied to quantify severity of knee osteoarthritis from magnetic-resonance imaging scans, again achieving significant correlation with an independent clinical assessment (*ρ* = 0.644, 95% C.I [0.585,0.696]).

## Introduction

In current clinical practice, assessment of impairment and disease severity typically relies on examinations by medical professionals^1,2^. As a result, assessment is often qualitative and its frequency is constrained by clinician availability. Developing data-driven quantitative metrics of impairment and disease severity has the potential to enable continuous and objective monitoring of patient recovery or decline. Such monitoring would facilitate personalized treatment and administration of appropriate therapeutic interventions in telehealth and other remotely supervised contexts where ongoing access to clinicians is not readily available^3–5^.

Artificial-intelligence (AI) models based on machine learning are a natural tool to perform data-driven patient assessment^6–19^. These models can be trained in a supervised fashion to estimate labels associated with patient data from large curated datasets of examples^11,14,20^. Unfortunately, it is often very challenging to assemble datasets containing an exhaustive representation of severity or impairment levels, which is necessary to ensure the accuracy of the AI models^21–26^. Moreover, supervised approaches require the existence of an objective quantitative metric that can be computed for every patient in the dataset, but such metrics do not exist for many medical conditions^27,28^.

To address these challenges, we consider the problem of performing automatic patient assessment using AI models trained *only on data from healthy subjects*. This is an anomaly detection problem, where the goal is to identify data points that are systematically different from a reference population^29^. Existing anomaly-detection methods for medical data are mostly based on generative models^30,31^. These models are designed to reconstruct high-dimensional data from a learned low-dimensional representation. Once trained, they are typically unable to accurately reconstruct data that are anomalous, due to their inconsistency with the training set. Consequently, the model reconstruction error tends to be higher for anomalies than for normal data, and can therefore be used as an anomaly-detection score. This approach has been applied to identify chronic brain infarcts^32^, Alzheimer’s disease^33^, microstructural abnormalities in diffusion MRI tractometry^34^, and abnormalities of cosmetic breast reconstruction in cancer patients^35^.

**Fig. 1:**
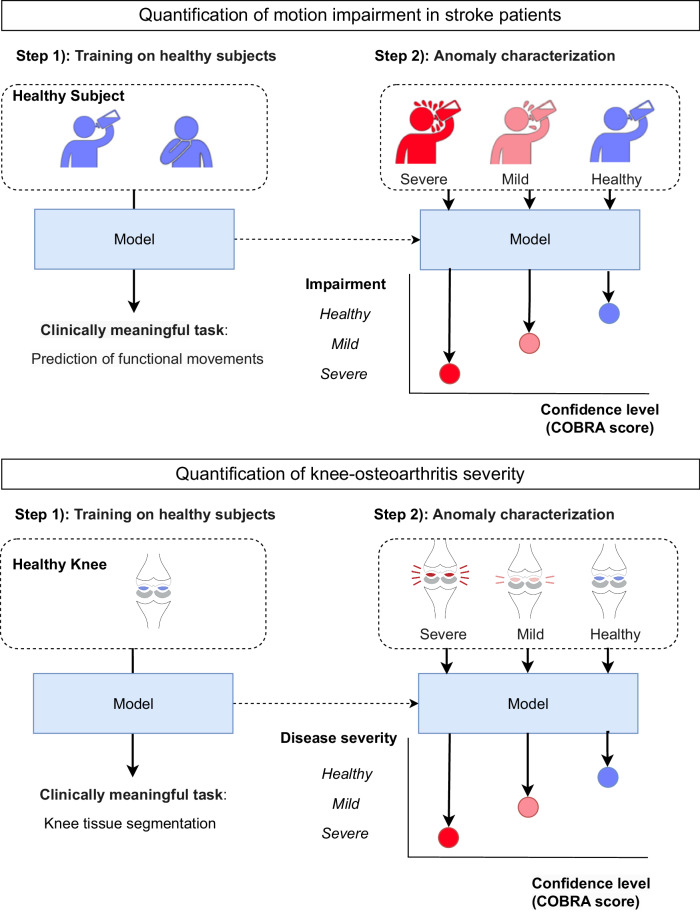
The COnfidence-Based chaRacterization of Anomalies (COBRA) score. In Step 1, an AI model is trained to perform a clinically meaningful task on data from healthy individuals. For impairment quantification in stroke patients, the task is prediction of functional primitive motions from videos or wearable-sensor data (top). For severity quantification of knee osteoarthritis, the task is segmentation of knee tissues from magnetic resonance imaging scans (bottom). In Step 2, the COBRA score is computed based on the confidence of the AI model when performing the task on patient data. Data from patients with higher degrees of impairment or severity differ more from the healthy population used for training, which results in decreased model confidence and hence a lower COBRA score.

Anomaly detection based on generative models has an important disadvantage: it does not constrain the AI model to learn clinically relevant features. Consequently, the model reconstruction error may depend on properties of the data unrelated to the medical condition of interest. Here, we propose a novel anomaly-detection framework that is *tailored to a specific medical condition*. This is achieved by utilizing an AI model that predicts an attribute of the data, which is directly relevant to the condition (e.g. type of motion primitive performed by the stroke-impaired side, or tissue type for knee osteoarthritis). Crucially, the model is trained exclusively on healthy subjects, using annotated data describing the attribute. When the models are presented with data where the attribute is affected by the medical condition of interest, we observe that the average model confidence tends to decrease proportionally to severity. This yields a quantitative patient-assessment metric, which we call the COnfidence-Based chaRacterization of Anomalies (COBRA) score. Figure 1 provides a schematic description of the proposed framework.

**Fig. 2:**
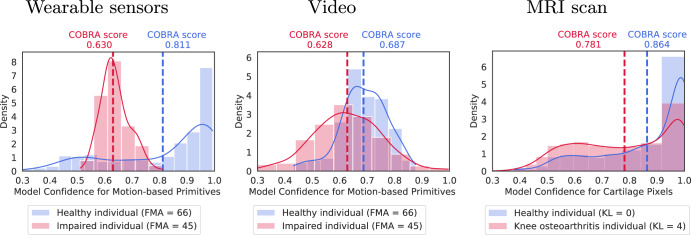
Averaging model confidence yields a discriminative subject-level metric. The plots show histograms and kernel density estimates of the confidence of a model trained on healthy subjects when presented with test data from an impaired or diseased patient (red), and from a held-out healthy individual (blue). The confidence distributions overlap, so individual values do not allow to discriminate between healthy and impaired individuals. In contrast, the average confidence is systematically higher for healthy subjects, and therefore provides a discriminative subject-level metric. The first and second plot correspond to wearable-sensor and video data associated with the same healthy and impaired individuals from the test cohort for quantification of stroke-induced impairment. The third plot corresponds to MRI scans from a healthy and diseased individual in the knee-osteoarthritis test cohort.

The COBRA score is inspired by a technique proposed in^36^, which identifies anomalous data points using the confidence of AI models. In this and subsequent works^37–41^ anomalies were identified based on the loss of confidence of the AI models for a *single data point*. The effectiveness of this approach depends on the overlap in the distribution of confidences^42^. In our applications of interest, AI models trained on healthy subjects tend to lose confidence *on average* when presented with multiple inputs from an impaired or diseased patient. However, the confidence for individual data points is very noisy and results in an unreliable metric, as illustrated by Fig. 2. For this reason, the COBRA score is computed using multiple data points for each subject, corresponding to different motions in the application to stroke and to different voxels in the application to knee osteoarthritis. Aggregating the confidence associated with multiple data via averaging dramatically reduces the noise, resulting in a stable and accurate subject-level metric.

Our proposed framework can be interpreted as a form of *normative modeling*, where the goal is to quantify individual deviations from a reference population^43,44^. Existing normative models in neuroscience and psychiatry are based on probabilistic regression, which explicitly captures the normal variation of brain-derived phenotypes^45^. In contrast, the AI models used to compute the COBRA score perform normative modeling implicitly, by learning features associated with the attribute of interest within the reference population.

We apply the COBRA score to automatically evaluate the impairment level of stroke patients. Stroke commonly causes motor impairment in the upper extremity (UE), characterized by loss of strength, precise control, and intrusive muscle co-activation, which collectively interfere with normal function. Rehabilitation seeks to reduce motor impairment through the repeated practice of functional movements with the UE. In this process, it is crucial to monitor the impairment level of the patient. The gold-standard method of measuring motor impairment is the Fugl-Meyer Assessment (FMA)^2^. Unfortunately, it requires in-person administration by a trained assessor and is time-consuming (30-45 minutes), which makes it impractical for frequent monitoring. Automatic assessment of motion impairment based on video or wearable-sensor data would address these limitations, facilitating actionable and granular tracking of motor recovery.

Motor impairment evaluation in stroke patients illustrates the difficulty of applying standard supervised AI methodology to patient assessment. An existing study shows the feasibility of the approach^46^, but only includes 17 patients. Training a supervised model to predict impairment and rigorously evaluating its performance on held-out data requires a database of at least hundreds, and ideally thousands of patients, labeled with the corresponding impairment level. However, the largest such publicly available dataset consists of just 51 patients^47^. Here, we use this dataset as a held-out test set to evaluate the proposed framework.

In order to assess impairment in stroke patients using the COBRA score, we trained AI models to predict classes of UE motion, known as functional primitives, from video and wearable-sensor data. The models were trained on a cohort of healthy individuals. Crucially, although the healthy cohort is relatively small (25 individuals), the number of labeled primitives per patient is large (typically around 300,000), which provides a rich training dataset with more than 6 million examples. Once trained on the healthy subjects, the models were applied to data from a test cohort of stroke patients and held-out healthy subjects performing nine different stroke rehabilitation activities. The confidence of the motion predictions for each test subject was averaged to compute the corresponding COBRA score. Our results show that the COBRA score is correlated with the Fugl-Meyer Assessment of the patients, obtained in person by trained experts, for both data modalities. The score is computed in under a minute and does not require expert input. This greatly expands on our preliminary findings, which used a similar approach with wearable-sensor data from a single rehabilitation activity^48^.

To demonstrate the general applicability of the COBRA framework, we show that it can be used to evaluate severity of knee osteoarthritis from magnetic resonance imaging (MRI) scans. Knee osteoarthritis is a musculoskeletal disorder characterized by a progressive loss of knee cartilage. To quantify severity, we trained an AI model to perform segmentation of different knee tissues (including cartilage) on MRIs of healthy knees. We then applied the model to knee MRIs from a test cohort of diseased patients and held-out healthy subjects. The confidence of the tissue predictions for each test subject was averaged to compute the corresponding COBRA score. The resulting COBRA score is again highly correlated with an independent assessment of disease severity (in this case, the Kellgren-Lawrence grade).

## Results

### Quantification of impairment in stroke patients

The application of the COBRA score to the impairment quantification in stroke patients was carried out using the publicly available StrokeRehab dataset^47^. StrokeRehab contains video and wearable-sensor data from a cohort of 29 healthy individuals and 51 stroke patients performing multiple trials of nine rehabilitation activities (described in Supplementary Tables 1, 2). The impairment level of each patient was quantified via the Fugl-Meyer assessment (FMA)^2^. The FMA score is a number between 0 (maximum impairment) and 66 (healthy) equal to the sum of itemized scores (each from 0 to 2) for 33 upper body mobility assessments carried out in-clinic by a trained expert. The wearable-sensor and video data are labeled to indicate what functional primitive is being performed by the paretic arm over time: reach (UE motion to make contact with a target object), reposition (UE motion to move into proximity of a target object), transport (UE motion to convey a target object in space), stabilization (minimal UE motion to hold a target object still), and idle (minimal UE motion to stand at the ready near a target object).

The COBRA score was computed based on AI models trained to predict the functional primitives performed by a training cohort, which includes 25 of the 29 healthy individuals (selected at random). The model input was either wearable sensor or video data. Detailed descriptions of these models are provided in the Methods section. The models were applied to a test cohort consisting of the remaining 4 healthy individuals and the 51 stroke patients. Demographic and clinical information about the training and test cohorts is provided in Table 1. The COBRA score is equal to the average of the model confidence for data points identified by the models as corresponding to functional primitives that involve motion (transport, reposition, and reach).

**Table 1:** Demographic and clinical characteristics of the training and testing cohorts for the application to quantification of motion impairment in stroke patients

	Training	Testing
Number of subjects	25	55
Trials	1265	2183
Age	62.4 ± 13.1	57.7 ± 14.0
Sex	13 male, 12 female	25 male, 30 female
Race^a^	10 W, 12 B, 0 A, 1 AI, 2 O	24 W, 14 B, 9 A, 0 AI, 8 O
Paretic Side	n/a	28 left, 23 right, 4 n/a
Fugl-Meyer Assessment	66	43.5 ± 16.2
Impairment level^b^	25 healthy	4 healthy, 20 mild, 23 moderate, 8 severe
Time since stroke	n/a	5.4 ± 6.1 years (for stroke patients)

The mean ± standard deviation is reported for age, Fugl-Meyer assessment and time since stroke.

^a^Race: White (W), Black (B), Asian (A), American Indian (AI), Other (O).

^b^Based on FMA: 0–25 is severe, 26–52 moderate, 53–65 mild, and 66 healthy.

**Fig. 3:**
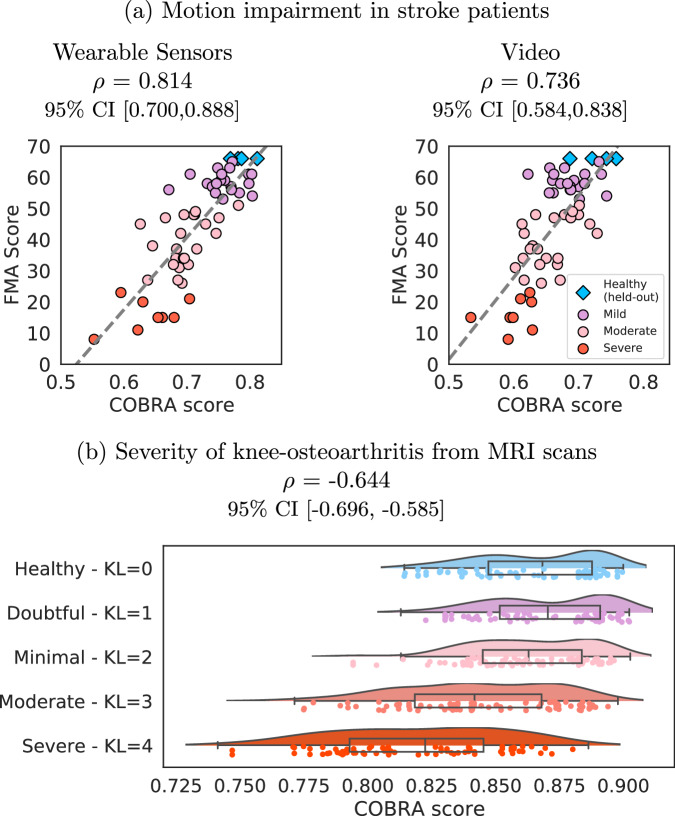
Correlation between the COBRA score and clinical assessment. **a** The graphs show scatterplots of the Fugl-Meyer assessment (FMA) score, based on in-person examination by an expert, and the proposed data-driven COBRA score computed from wearable-sensor data (left) and from video data (right). The correlation coefficient *ρ* between the two scores is high, particularly for the wearable-sensor data. **b** The graph shows scatterplots and density plots of COBRA scores computed from magnetic-resonance imaging (MRI) knee scans of patients with different Kellgren-Lawrence (KL) grades. The KL grade and the COBRA score exhibit significant inverse correlation.

The COBRA score was evaluated by computing its Pearson correlation coefficient with the Fugl-Meyer Assessment (FMA) score^2^ on the test cohort of 51 stroke patients and 4 healthy individuals (*n* = 55). The correlation coefficient is 0.814 (95% CI [0.700,0.888]) for the wearable-sensor data and 0.736 (95% CI [0.584, 0.838]) for the video data. Figure 3 (a) shows scatterplots of the COBRA and FMA scores. For both data modalities, the COBRA score has a strong, statistically significant correlation with the in-clinic assessment. The Supplementary Methods reports additional results on the wearable-sensor data using a completely different AI architecture for primitive prediction. The correlation coefficient between the resulting COBRA score and the FMA score is again high: 0.774 (95% CI [0.636, 0.865]). This indicates that the proposed approach is robust to the choice of underlying AI model.

**Fig. 4:**
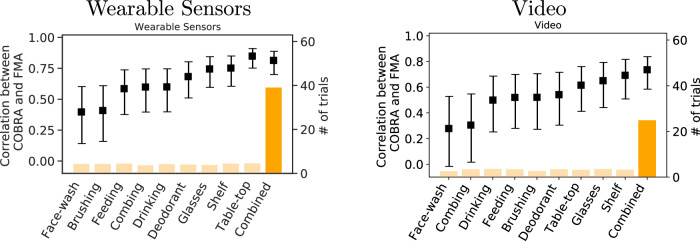
Impairment quantification from individual rehabilitation activities. The graph shows the correlation coefficient (indicated by black markers with 95% confidence intervals) between the Fugl-Meyer score of stroke patients, and the COBRA score computed from single activities using wearable-sensor (left) or video (right) data. The number of trials available for each activity are indicated by the yellow bars. Simple, more structured activities (Glasses, Shelf, Table-top) have higher correlation than more complicated activities (Face-wash, Feeding, Combing) for both data modalities.

Figure 4 reports the correlation coefficients between the FMA score and the COBRA score computed using subsets of the data corresponding to individual rehabilitation activities (see Supplementary Tables 1, 2 for a detailed description of the activities). Scatterplots of the FMA and COBRA scores for each activity are provided in Supplementary Figs. 2, 3. For both data modalities, the correlation is higher for more structured activities (moving objects to targets on a table-top or shelf, donning glasses) and is lower for more complex activities (hair-combing, face-washing, teeth-brushing, feeding), which tend to involve more heterogeneous motions across individuals. The correlation coefficient with the FMA score is lower for the COBRA score computed from individual activities than for the COBRA score that aggregates all activities. The only exception is the table-top task, which is the most regular and structured activity. The correlation between the corresponding COBRA score, computed from wearable-sensor data, and the FMA score is very high (0.849, 95% CI [0.752, 0.910]), which suggests that it may be possible to obtain accurate impairment assessment from a reduced number of data using activities that are highly structured.

**Fig. 5:**
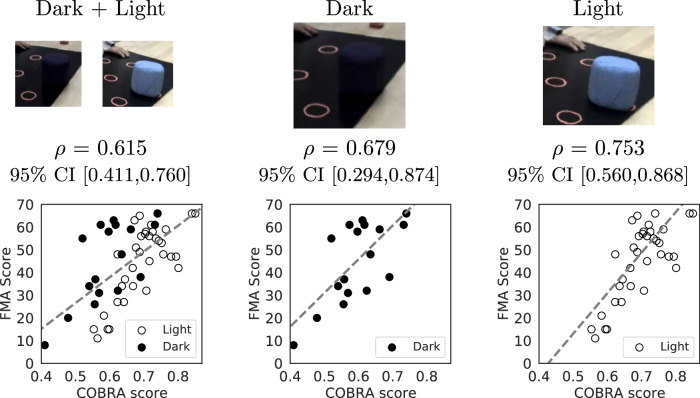
Object color as a confounding factor for the video-based COBRA score and correction via stratification. The table-top rehabilitation activity in the stroke impairment quantification task involves dark and light-colored objects (top row). The bottom left scatterplot shows the COBRA score computed only using video data from this activity and the corresponding Fugl-Meyer assessment (FMA) score. The dark objects are difficult to detect, which results in a systematic loss of confidence in the video-based AI model, and hence lower COBRA scores (independently from the FMA score). The bottom middle and right scatterplots show that stratifying according to object color corrects for the confounding factor, improving the correlation coefficient *ρ* between the COBRA and FMA scores.

An important consideration when applying the proposed framework is that extraneous factors may produce a spurious decrease in the confidence of the AI model. Figure 5 shows that this occurs for the table-top activity, which was carried out with light-colored and dark-colored objects by different subjects. Dark objects are much more difficult to detect in videos, which produces a systematic loss of confidence in the video-based AI model that translates to lower COBRA scores. This explains why the correlation between the FMA score and the COBRA score is lower for the table-top video data than for the table-top wearable-sensor data, which is unaffected by this confounding factor. As depicted in Fig. 5, we can correct for the confounding factor by stratifying the subjects according to the object color. This increases the COBRA score from 0.615 (95% CI [0.411, 0.760]) to 0.679 (95% CI [0.294, 0.874]) for dark objects and 0.756 (95% CI [0.553, 0.874]) for light objects. For comparison, the correlation of the video-based COBRA score computed from all activities is 0.736 (95% CI [0.584,0.838]). Supplementary Fig. 4 shows that image quality can also act as a confounding factor: blurring the video images results in a systematic decrease of the COBRA score, which can also be corrected via stratification.

**Fig. 6:**
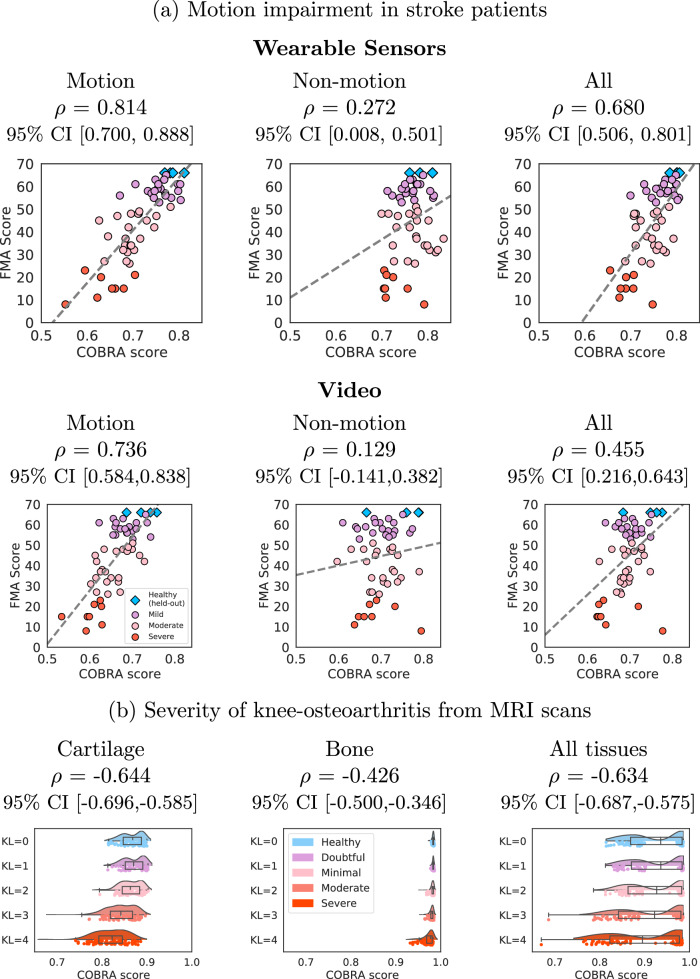
The COBRA score exploits clinically-relevant structure. **a** The first row show scatterplots of the clinical Fugl-Meyer assessment and the proposed COBRA score, obtained from wearable-sensor data. In the left graph, the COBRA score is computed only using data identified as clinically relevant (i.e. corresponding to motion actions). In the middle graph, the score is computed using the remaining data. In the right graph, it is computed using all of the data. The second row shows the same scatterplots, with the only difference that the COBRA score is obtained from video data. The COBRA score based on clinically-relevant data achieves a higher correlation with the clinical assessment in both cases. **b** The graphs show scatterplots of the Kellgren-Lawrence grade and the proposed COBRA score, obtained from knee MRI scans. In the left graph, the COBRA score is computed only using data identified as clinically relevant (i.e. corresponding to cartilage tissue). In the middle graph, the score is computed using the remaining data. In the right graph, it is computed using all of the data. The COBRA score using clinically relevant data again achieves a higher correlation with the clinical assessment.

The COBRA score is the average of the AI-model confidence for data points identified by the model as corresponding to functional actions that involve motion (*reach*, *reposition*, *transport*), as opposed to functional actions that do not (*idle*, *stabilize*). These data can be considered as *clinically relevant* to impairment quantification associated with motion. Figure 6 shows that the correlation coefficient between the FMA score and a COBRA score computed from data points identified as non-motion functional actions is low (in fact, for the video data it is not even statistically significant). It also shows that a COBRA score computed from all actions has a lower correlation with the FMA score than the proposed motion-based COBRA score for both data modalities.

### Quantification of knee-osteoarthritis severity

The application of the COBRA score to the quantification of knee-osteoarthritis (OA) severity was carried out using the publicly available OAI-ZIB dataset^49^. This dataset provides 3D MRI scans of 101 healthy right knees and 378 right knees affected by knee osteoarthritis, a long-term degenerative joint condition. Each knee is labeled with the corresponding Kellgren-Lawrence (KL) grade^50^, retrieved from the NIH Osteoarthritis Initiative collection^51^. The KL grade quantifies OA severity on a scale from 0 (healthy) to 4 (severe), as illustrated in Supplementary Fig. 1. Each voxel in the MRI scans is labeled to indicate the corresponding tissue (*tibia bone*, *tibia cartilage*, *femur bone*, *femur cartilage* or *background*).

The COBRA score was computed based on an AI model trained to perform tissue segmentation on a training cohort of 44 healthy individuals (selected at random). A detailed description of the model is provided in the Methods section. The model was applied to a test cohort consisting of the remaining 57 healthy individuals and the 378 patients with knee OA. Demographic and clinical information about the training and test cohorts is provided in Table 2. The COBRA score is equal to the average of the model confidence for data points identified by the model as corresponding to cartilage tissue (*tibia cartilage* and *femur cartilage*).

**Table 2:** Demographic and clinical characteristics of study participants for the application to quantification of knee-osteoarthritis severity

	Training	Testing
Number of individuals	44	435
Age	59.2 ± 8.2	62.0 ± 9.4
Sex	20 male, 24 female	228 male, 207 female
Race^a^	36 W, 7 B, 1 O	339 W, 81 B, 5 A, 10 O
Kellgren-Lawrence grades	44 healthy (KL = 0)	57 healthy (KL = 0), 58 doubtful (KL = 1), 109 minimal (KL = 2), 138 moderate (KL = 3), 73 severe (KL = 4)

The mean ± standard deviation is reported for age.

^a^Race: White (W), Black (B), Asian (A), Other (O).

The COBRA score was evaluated by computing its Pearson correlation coefficient with the Kellgren-Lawrence (KL) grading scores^50^ on the test cohort of 378 patients with knee OA and 57 healthy subjects (n=435), which equals –0.644 (95% CI [–0.696, –0.585]). There is therefore a significant inverse correlation between the scores, indicating that the COBRA score quantifies knee OA severity. Figure 3(b) shows scatterplots and density plots of the COBRA scores corresponding to different KL grades. The Supplementary Methods section reports additional results using a different AI architecture for tissue segmentation. The magnitude of the correlation coefficient between the resulting COBRA score and the KL grade is lower, but still statistically significant: –0.429 (95% CI [–0.503,–0.349]).

The COBRA score is computed as an average of the AI-model confidence for voxels identified by the model as corresponding to cartilage, as opposed to bone tissue. These data can be considered as *clinically relevant* because knee OA produces gradual degradation of articular cartilage (bone alterations and osteophyte formation may also occur, but are less frequent)^52,53^. Figure 6 shows that the magnitude of the correlation coefficient between the KL score and the COBRA score is significantly lower than for cartilage. The magnitude of the correlation coefficient for the COBRA score computed from all voxels is only slightly lower than that of the proposed cartilage-based COBRA score, indicating that including bone is not very detrimental.

## Discussion

In this work we introduce the COBRA score, a data-driven anomaly-detection framework for automatic quantification of impairment and disease severity. We show its utility for clinically relevant quantification in two different medical conditions (stroke and knee osteoarthritis) and for three different data modalities (wearable sensors, video and MRI). The framework is suitable for applications where it is challenging to gather large-scale databases of patients with different degrees of impairment or severity, because it only requires data from a healthy cohort of moderate size. The domains of potential applicability are broad, as they encompass any condition affecting patient motion, as in our application to stroke, or producing structural abnormalities in imaged tissues, as in our application to knee osteoarthritis.

From a methodological perspective, our results suggest that fine-grained annotations describing clinically relevant attributes can be useful *even if they are only available for healthy subjects*. We hypothesize that AI models trained with such annotations can be leveraged in different ways beyond the proposed approach. To illustrate this, an alternative anomaly-detection procedure that does not utilize model confidence is included in the Supplementary Methods section.

Our study identifies a key consideration when applying the proposed framework: confounding factors unrelated to the medical condition of interest (e.g. object color or blurriness in a video) can influence the confidence of the AI models, and therefore distort the COBRA score. This is an instance of a general challenge inherent to the use of deep neural networks: these models are so flexible that they can easily learn spurious structure in high-dimensional data^42,54^. Our results suggest that the influence of confounding factors can be mitigated by gathering a training set of healthy subjects that is sufficiently diverse with respect to the population of interest. In the case of stroke-induced impairment, we show that this can be achieved by utilizing multiple different rehabilitation activities. In addition, we demonstrate that it is possible to explicitly correct for known confounding factors via stratification. These factors could be identified by monitoring their correlation with the average confidence of the AI models over multiple individuals (under the assumption that the factors are uncorrelated with impairment or disease severity). Nevertheless, automatic identification and control of confounders is an important topic for future research.

## Methods

In this section we describe a general framework to estimate impairment and disease severity using AI models trained only on data from healthy subjects. We frame this as an anomaly detection and quantification problem, where the goal is to identify subjects that deviate from the healthy population, and to quantify the extent of this deviation.

### Confidence-based characterization of anomalies

The proposed COnfidence-Based chaRacterization of Anomalies (COBRA) framework utilizes a model trained to perform an AI task only on healthy patients. Intuitively, if the model has low confidence when performing the task on a new subject, this indicates that the subject deviates from the healthy population. In order to ensure that this deviation is due to a certain type of impairment or disease, it is crucial to choose an appropriate AI task. For quantification of stroke-induced impairment, we predict the functional actions carried out by the subject from wearable sensor or video data. For the application to knee osteoarthritis, we predict the tissue present in each voxel of a 3D MRI scan.

Let us assume that we have access to a training cohort of *N*_train_ healthy subjects, and that each of them is associated with a set of annotated data relevant to the medical condition of interest:

1 $$\mathrm{T}_{i}:= \left\{\left({x}_{1}^{[i]},{y}_{1}^{[i]}\right),\ldots ,\left({x}_{{M}_{i}}^{[i]},{y}_{{M}_{i}}^{[i]}\right)\right\},\quad 1\le i\le {N}_{{{\mathrm{train}}}\,}.$$

Here ${x}_{j}^{[i]}\in {{\mathbb{R}}}^{L}$ denotes the *j*th data point associated with the *i*th subject, and *M*_*i*_ is the number of data available for that subject. The label ${y}_{j}^{[i]}\in \left\{1,\ldots ,K\right\}$ assigns ${x}_{j}^{[i]}$ to one of *K* predefined classes. For the stroke application, the label encodes the functional action carried out by the subject at a certain time. The corresponding data point is a segment of wearable-sensor or video data. For the knee-osteoarthritis application, the label encodes the type of tissue at a certain position in the knee, and the corresponding data are the surrounding MRI voxels.

The training dataset

2 $$\mathrm{S}_{{{\mathrm{train}}}\,}:= \left\{\mathrm{T}_{1},\ldots ,\mathrm{T}_{{N}_{{{\mathrm{train}}}\,}}\right\}$$

is used to train an AI model $f:{{\mathbb{R}}}^{L}\to {[0,1]}^{K}$ to predict the labels from the data. The input to the model is an *L*-dimensional data point and the output is a *K*-dimensional vector

3 $${p}_{j}^{[i]}:= f({x}_{j}^{[i]}),\quad 1\le i\le {N}_{{{\mathrm{train}}}\,},\ 1\le i\le {M}_{i},$$

where the *k*th entry is an estimate of the probability that the data point belongs to the *k*th class. In our applications of interest, the models are deep neural networks, described in detail below. Crucially, if the dataset associated with each subject is large, then the total number of training examples

4 $${M}_{{{\mathrm{train}}}\,}:= \sum\limits_{i=1}^{{N}_{{{\mathrm{train}}}\,}}{M}_{i}$$

is orders of magnitude larger than the number of training subjects *N*_train_. This enables us to train deep-learning models using relatively small training cohorts.

Let ${X}_{{{\mathrm{test}}}\,}:= \{{x}_{1}^{{{\mathrm{test}}}\,},\ldots ,{x}_{{M}_{{{\mathrm{test}}}\,}}^{{{\mathrm{test}}}\,}\}$ denote a dataset associated with a test subject. We can obtain probabilities corresponding to the *j*th test data point by applying the trained AI model,

5 $${p}_{j}^{{{\,\mathrm{test}}}\,}:= f({x}_{j}^{{{\mathrm{test}}}\,}),\quad 1\le j\le {M}_{{{\mathrm{test}}}\,}.$$

This yields a prediction of the class associated with the data point

6 $$z^{\,\rm{test}}_j:= \arg \mathop{\max}\limits_{1 \leq k \leq K} p^{\,\rm{test}}_j[k],$$

where ${p}_{j}^{{{\mathrm{test}}}\,}[k]$ denotes the *k*th entry of ${p}_{j}^{{{\mathrm{test}}}\,}$. The estimated probability that the data point belongs to the predicted class is commonly known as the *confidence* of the model (see e.g.^55^),

7 $$c^{\operatorname{test}}_j := \mathop{\max}\limits_{1 \leq k \leq K} p^{\,\rm{test}}_j[k],$$

because it can be interpreted as an estimate of the probability that the model prediction is correct.

Several existing works propose to use confidence values to perform anomaly detection^36–41^. Intuitively, if a model is well trained (and there is no inherent uncertainty in the training labels^56^), it should be able to confidently classify new examples. Therefore low model confidence is evidence that the data point may be anomalous, in the sense that it deviates from the training distribution. Our proposed framework builds upon this idea, incorporating two novel elements. First, multiple data points are aggregated to perform subject-level anomaly detection. As illustrated by Fig. 2, this is critical to achieve accurate anomaly detection in our applications of interest, because the individual confidences are very noisy. Second, we determine which of the classes are most clinically relevant, and restrict our attention to data points predicted to belong to those classes. As reported in Fig. 6, for the stroke application this provides a substantial improvement over using all the data.

Let ${{{\mathrm{CR}}}}\subseteq \left\{1,\ldots ,K\right\}$ denote the subset of clinically relevant classes, and

8 $${{{{\mathrm{J}}}}}_{{{\mathrm{relevant}}}\,}:= \left\{j:{z}_{j}^{{{\mathrm{test}}}\,}\in {{{\mathrm{CR}}}}\right\}$$

the subset of test data predicted to belong to those classes. We define the COBRA score as the arithmetic average of the confidences associated with the data in ${{{{\mathrm{J}}}}}_{{{\mathrm{relevant}}}\,}$,

9 $${{\mathrm{COBRA}}}\,({X}_{{{\mathrm{test}}}\,}):= \frac{1}{| {{{{\mathrm{J}}}}}_{{{\mathrm{relevant}}}\,}| }\sum\limits_{j\in {{{{\mathrm{J}}}}}_{{{\mathrm{relevant}}}\,}}{c}_{j}^{{{\mathrm{test}}}\,}.$$

The lower the COBRA score, the less confident the AI model is on average when performing the task on the test subject, which indicates a greater degree of impairment or disease.

### Estimation of stroke-related motor impairment

In order to apply the COBRA framework to automatic impairment quantification in stroke patients, we propose to utilize auxiliary AI models trained to predict the functional primitive carried out by the subjects’ paretic upper extremity (UE) while performing rehabilitation activities. The *K* ≔ 5 primitive classes are *reach*, *reposition*, *transport*, *stabilize*, and *idle*. UE motor impairment affects the three functional primitives involving motion

10 $${{{\mathrm{CR}}}}:= \left\{\text{transport},\,\text{reposition},\,\text{reach}\right\},$$

rendering them systematically different to those of healthy individuals. Our hypothesis is that this causes AI models trained on healthy subjects to lose confidence when they are applied to stroke patients, and that the loss of confidence is indicative of the degree of impairment. In the following paragraphs, we describe the AI models that we use to test this hypothesis for two different data modalities, wearable sensors and video.

The wearable-sensor data is a 77-dimensional time series, recorded at 100 Hz using nine inertial measurement units (IMUs) attached to the upper body^47^. The data correspond to kinematic features of 3D linear accelerations, 3D quaternions, joint angles from the upper body, and a binary value that indicates the side (left or right) performing the motion. In order to identify functional primitives from these data, we utilized a Multi-Stage Temporal Convolutional Network (MS-TCN)^57^. This model was found to be effective for primitive segmentation in a prior study^47^. In the Supplementary Methods section we report results with a different model architecture, based on a sequence-to-sequence model^47,58^.

MS-TCN is a state-of-the-art deep-learning model for action segmentation consisting of four convolutional stages, each composed of 10 layers of dilated residual convolutions with 64 output channels. A softmax layer at the end of the network produces the final output, which is a 5-dimensional vector indicating the probability that each entry in the time series corresponds to each functional primitive. The model was trained on the healthy training cohort using the weighted cross-entropy loss function proposed in^59^. This cost function was minimized for 50 epochs using the Adam optimizer^60^ with a learning rate of 5 ⋅ 10^−3^ (selected via cross-validation). The accuracy and precision of the resulting model are reported in Supplementary Table 4.

The video data were acquired with two high-speed (60 Hz), high-definition (1088 × 704 resolution) cameras (Ninox, Noraxon) positioned orthogonally <2 m from the subject. The cameras have a focal length of f4.0 mm and a large viewing window (length: 2.5 m, width: 2.5 m). The videos were then downsampled to a resolution of 256 × 256 to enable efficient processing. To perform functional primitive identification from these data, we utilized the X3D model^61^, a 3D convolutional neural network designed for primitive classification from video data. The model was pretrained on the Kinetic dataset^62^, where the labels are high-level activities such as running, climbing, sitting, etc.

Following the approach proposed in^47^, after pretraining, the X3D model was fine-tuned to perform classification of functional primitives on the rehabilitation activities performed by the healthy training cohort. The input to the model are video segments with duration two seconds, as suggested in^63^, and the output is the estimated probability that the central frame corresponds to each of the five functional primitives. Model fine-tuning was carried out by minimizing the cross entropy between these probabilities and the functional primitive labels via stochastic gradient descent with a base learning rate of 0.01 and a cosine learning rate policy. The accuracy and precision of the resulting model on held-out subjects are reported in the Supplementary Tables 3, 4.

### Estimation of knee-osteoarthritis severity

In order to apply the COBRA framework to automatic quantification of knee-osteoarthritis severity we propose to utilize an auxiliary AI model trained to predict the type of tissue in each voxel of a 3D MRI scan. The *K* ≔ 5 classes for this classification problem are *femur bone*, *femur cartilage*, *tibia bone*, *tibia cartilage* and *background* (indicating absence of tissue). Knee osteoarthritis deforms cartilage structure, so the clinically relevant labels are chosen to be

11 $${{\mathrm{CR}}}:= \left\{{\text{femur}}\,{\text{cartilage}},\,{\text{tibia}}\,{\text{cartilage}}\right\}.$$

Our hypothesis is that the systematic difference in cartilage structure causes AI models trained on healthy knees to lose confidence when applied to diseased knees, and that the loss of confidence is indicative of disease severity.

In order to predict tissue type we applied a Multi-Planar U-Net^64^. In the Supplementary Methods section, we report results with a different model architecture, based on a 3D U-Net^65^. The Multi-Planar U-Net processes the input 3D MRI scan from different views using a version of the 2D U-Net architecture^66^. The output from the different views are then averaged to produce a probability estimate at each 3D voxel. During training, random elastic deformations (RED) are applied to a third of the images in each batch to improve generalization^64^.

The model was trained by minimizing the cross entropy loss between the estimated probabilities and the 3D voxel-wise labels corresponding to 37 of the 44 healthy individuals in the training cohort. The remaining 7 individuals were used as a validation set. In the cost function, images augmented via RED were downweighted by a factor of 1/3. The Adam optimizer was used for minimization, with an initial learning rate of 5 ⋅ 10^−5^ that was reduced by 10% after two consecutive epochs without improvement in the validation Dice score. A criterion based on the validation Dice score (excluding background) was used to perform early stopping. Additional hyperparameters are listed in Supplementary Tables of^64^. The accuracy and precision of the resulting model are reported in Supplementary Table 7.

### Ethics statement

For the StrokeRehab dataset, all subjects provided written informed consent in accordance with the Declaration of Helsinki. The study was approved by the Institutional Review Board at the New York University Grossman School of Medicine.

For the OAI-ZIB dataset, the National Institute of Arthritis and Musculoskeletal and Skin Diseases (NIAMS) at the National Institutes of Health (NIH) appointed an independent Observational Study Monitoring Board (OSMB) to oversee the Osteoarthritis Initiative (OAI) study from 2002 to 2014. The OSMB was disbanded upon study completion when monitoring obligations were fulfilled.

### Reporting summary

Further information on research design is available in the Nature Research Reporting Summary linked to this article.

### Supplementary information

Supplementary Information

Reporting Summary

## Data Availability

Links to all the data used in this study are available at https://github.com/fishneck/COBRA/tree/main/data.

## Code availability

Code to reproduce all results is available at https://github.com/fishneck/COBRA.
