# Supplementary material for: Quantifying impairment and disease severity using AI models trained on healthy subjects
Source: NPJ Digit Med. 2024 Jul 6;7:180. doi: 10.1038/s41746-024-01173-x (PMC11226623; doi:10.1038/s41746-024-01173-x)
Supplement: Supplementary file 1 — Supplementary Information [file 41746_2024_1173_MOESM1_ESM.pdf]

1 Supplementary Tables

Supplementary Table 1 Description of the activities performed by the subjects in the dataset used for quantification of stroke-induced impairment (1/2).

Activity	Workspace	Target object(s)	Instructions
Face-wash	Sink with a small tub (32.3 x 24.1 x 2.5 cm^3) in it and two folded washcloths on either side of the countertop, 30 cm from edge closest to patient	Washcloths, faucet handle, and tub	Fill tub with water, dip washcloth on the right side into water, wring it, wiping each side of their face with wet washcloth, place it back on countertop. Use washcloth on the left side to dry face, place it back on countertop
Deodorant	Tabletop with deodorant placed at midline, 25 cm from edge closest to patient	Deodorant (solid twist-base)	Remove cap, twist base a few times, apply deodorant, replace cap, untwist the base, put deodorant on table
Combing	Tabletop with comb placed at midline, 25 cm from edge closest to patient	Comb	Pick up comb and comb both sides of head
Glasses	Tabletop with glasses placed at midline, 25 cm from edge closest to patient	Pair of glasses	Wear glasses, return hands to table, remove glasses and place on table
Feeding	Table top with a standard-size paper plate (21.6 cm diameter) placed at midline, 2 cm from edge, utensils placed 3 cm from edge, 5 cm from either side of plate, a baggie with a slice of bread placed 25 cm from edge, 23 cm left of midline, and a margarine packet placed 32 cm from edge, 17 cm right of midline	Paper plate, fork, knife, re-sealable sandwich baggie, slice of bread, single-serve margarine container	Remove bread from plastic bag and put it on plate, open margarine pack and spread it on bread, cut bread into four pieces, cut off and eat a small bite-sized piece

Supplementary Tables 1 and 2 provide a detailed description of the rehabilitation activities carried out by the subjects in the dataset used for quantification of stroke-induced impairment.

Supplementary Tables 3, 4, 5 and 6 report the accuracy and precision of the AI models for stroke functional-primitive prediction described in the Methods section.

Supplementary Table 7 reports the voxel-wise accuracy and precision of the AI model for segmentation of MRI scans described in the Methods section.

Supplementary Table 2 Description of the activities performed by the subjects in the dataset used for quantification of stroke-induced impairment (2/2).

Activity	Workspace	Target object(s)	Instructions
Drinking	Tabletop with water bottle and paper cup 18 cm to the left and right of midline, 25 cm from edge closest to patient	Water bottle (12 oz), paper cup (4 oz)	Open water bottle, pour water into cup, take a sip of water, place cup on table, and replace cap on bottle
Brushing	Sink with toothpaste and toothbrush on either side of the countertop, 30 cm from edge closest to patient	Travel-sized toothpaste, toothbrush with built-up foam grip, faucet handle	Wet toothbrush, apply toothpaste to toothbrush, replace cap on toothpaste tube, brush teeth, rinse toothbrush and mouth, place toothbrush back on countertop
Table-top	Horizontal circular array (48.5 cm diameter) of 8 targets (5 cm diameter)	Toilet paper roll wrapped in self-adhesive wrap	Move the roll between the center and each outer target, resting between each motion and at the end
Shelf	Shelf with two levels (33 cm and 53 cm) with 3 targets on both levels (22.5 cm, 45 cm, and 67.5 cm away from the left-most edge)	Toilet paper roll wrapped in self-adhesive wrap	Move the roll between the center target and each target on the shelf, resting between each motion and at the end

Supplementary Table 3 Performance of the AI model used to compute the COBRA score for quantification of stroke-induced impairment from wearable-sensor data on held-out healthy subjects. The models predict the functional primitive from the input data. There are five primitives, three of which are associated with motion. The motion-based accuracy/precision is calculated by averaging the corresponding metric over the motion primitives. The overall accuracy/precision is the average over all primitives. 95% CIs are shown in brackets.

Activity	Motion-based Accuracy	Motion-based Precision	Overall Accuracy	Overall Precision
All	0.734 [0.726,0.747]	0.781 [0.773,0.791]	0.755 [0.747,0.761]	0.719 [0.712,0.726]
Brushing	0.588 [0.574,0.602]	0.613 [0.598,0.625]	0.661 [0.652,0.672]	0.616 [0.605,0.624]
Combing	0.815 [0.803,0.826]	0.804 [0.794,0.816]	0.763 [0.750,0.777]	0.735 [0.725,0.746]
Deodorant	0.692 [0.681,0.702]	0.693 [0.682,0.705]	0.718 [0.708,0.726]	0.682 [0.674,0.691]
Drinking	0.696 [0.686,0.708]	0.740 [0.729,0.748]	0.743 [0.736,0.751]	0.710 [0.703,0.716]
Face-wash	0.605 [0.591,0.616]	0.628 [0.618,0.638]	0.585 [0.572,0.593]	0.569 [0.561,0.576]
Feeding	0.623 [0.608,0.642]	0.643 [0.625,0.662]	0.676 [0.662,0.688]	0.653 [0.642,0.665]
Glasses	0.781 [0.773,0.790]	0.768 [0.759,0.775]	0.699 [0.690,0.709]	0.686 [0.678,0.693]
Shelf	0.831 [0.822,0.838]	0.927 [0.922,0.932]	0.835 [0.827,0.843]	0.773 [0.765,0.779]
Table-top	0.807 [0.801,0.813]	0.874 [0.868,0.879]	0.746 [0.736,0.759]	0.716 [0.710,0.723]

Supplementary Table 4 Performance of the AI model used to compute the COBRA score for quantification of stroke-induced impairment from wearable-sensor data on subjects with different levels of impairment. Performance degrades as the impairment level increases. The metrics are defined as in Supplementary Table 3. 95% CIs are shown in brackets.

Impairment Level[1]	Motion-based Accuracy	Motion-based Precision	Overall Accuracy	Overall Precision
Healthy	0.747 [0.738,0.757]	0.773 [0.763,0.783]	0.753 [0.743,0.760]	0.725 [0.716,0.732]
Mild	0.707 [0.696,0.716]	0.689 [0.678,0.697]	0.674 [0.666,0.681]	0.669 [0.661,0.676]
Moderate	0.541 [0.532,0.551]	0.536 [0.535,0.551]	0.545 [0.539,0.552]	0.570 [0.562,0.578]
Severe	0.354 [0.342,0.367]	0.333 [0.321,0.346]	0.373 [0.364,0.383]	0.395 [0.386,0.405]

[1]Based on Fugl-Meyer Assessment: 0-25 is severe, 26-52 is moderate, 53-65 is mild, and 66 is healthy.

Supplementary Table 5 Performance of the AI model used to compute the COBRA score for quantification of stroke-induced impairment from video data on held-out healthy subjects. The metrics are defined as in Supplementary Table 3. 95% CIs are shown in brackets.

Activity	Motion-based Accuracy	Motion-based Precision	Overall Accuracy	Overall Precision
All	0.732 [0.720,0.740]	0.654 [0.643,0.663]	0.608 [0.597,0.616]	0.661 [0.650,0.672]
Brushing	0.781 [0.766,0.795]	0.709 [0.692,0.720]	0.611 [0.599,0.622]	0.670 [0.656,0.680]
Combing	0.714 [0.706,0.724]	0.817 [0.803,0.831]	0.547 [0.540,0.556]	0.631 [0.623,0.639]
Deodorant	0.629 [0.618,0.643]	0.512 [0.497,0.529]	0.443 [0.435,0.453]	0.576 [0.562,0.590]
Drinking	0.619 [0.605,0.633]	0.542 [0.527,0.557]	0.493 [0.482,0.502]	0.539 [0.526,0.551]
Face-wash	0.648 [0.635,0.661]	0.645 [0.633,0.660]	0.469 [0.461,0.478]	0.560 [0.551,0.573]
Feeding	0.524 [0.502,0.541]	0.421 [0.399,0.446]	0.467 [0.454,0.478]	0.494 [0.477,0.508]
Glasses	0.708 [0.699,0.715]	0.673 [0.399,0.446]	0.513 [0.454,0.478]	0.667 [0.477,0.508]
Shelf	0.717 [0.709,0.726]	0.633 [0.624,0.642]	0.556 [0.506,0.519]	0.612 [0.568,0.655]
Table-top	0.768 [0.760,0.777]	0.693 [0.683,0.702]	0.614 [0.605,0.623]	0.620 [0.603,0.637]

Supplementary Table 6 Performance of the AI model used to compute the COBRA score for quantification of stroke-induced impairment from video data on subjects with different levels of impairment. Performance degrades as the impairment level increases. The metrics are defined as in Supplementary Table 3. 95% CIs are shown in brackets.

Impairment Level[1]	Motion-based Accuracy	Motion-based Precision	Overall Accuracy	Overall Precision
Healthy	0.732 [0.722,0.742]	0.654 [0.643,0.664]	0.608 [0.599,0.618]	0.662 [0.652,0.672]
Mild	0.615 [0.605,0.627]	0.501 [0.492,0.512]	0.505 [0.497,0.513]	0.551 [0.542,0.561]
Moderate	0.555 [0.546,0.566]	0.421 [0.411,0.432]	0.441 [0.433,0.448]	0.496 [0.487,0.505]
Severe	0.425 [0.410,0.439]	0.348 [0.338,0.356]	0.352 [0.341,0.362]	0.339 [0.330,0.348]

[1]Based on Fugl-Meyer Assessment: 0-25 is severe, 26-52 is moderate, 53-65 is mild, and 66 is healthy.

Supplementary Table 7 Voxel-wise performance of the AI models used to compute the COBRA score for quantification of knee-osteoarthritis severity from MRI scans on held-out healthy subjects. 95% CIs are shown in brackets.

Group	Accuracy	Precision
KL = 0 (Healthy)	0.995 [0.993,0.997]	0.922 [0.878,0.952]
KL = 1 (Doubtful)	0.994 [0.992,0.996]	0.922 [0.890,0.956]
KL = 2 (Minimal)	0.994 [0.990,0.996]	0.907 [0.852,0.945]
KL = 3 (Moderate)	0.992 [0.983,0.996]	0.885 [0.816,0.931]
KL = 4 (Severe)	0.991 [0.979,0.995]	0.851 [0.721,0.917]

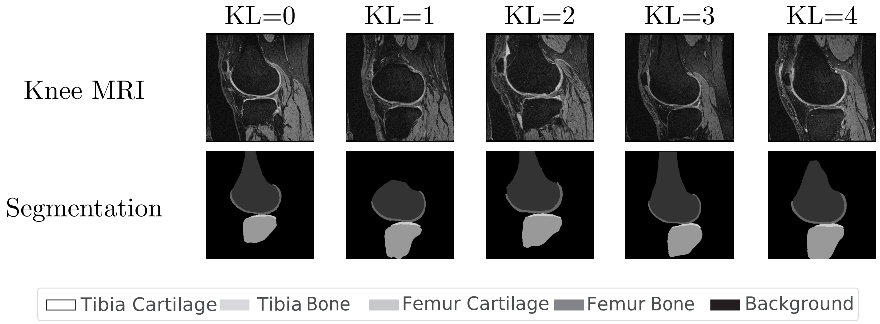

Supplementary Figure 1 Knee MRI images for subjects with different Kellgren-Lawrence (KL) grades (top) and corresponding segmentation annotations (bottom) indicating the tissue in each voxel.

2 Supplementary Figures

Supplementary Figure 1 shows examples of the MRI scans used for quantification of knee-osteoarthritis severity.

Supplementary Figures 2, and 3 show scatterplots of the FMA and COBRA scores for each rehabilitation activity.

In the main article (Results section, Figure 5), we show that object color is a confounding factor, which can spuriously reduce model confidence and therefore distort the COBRA score. To complement this observation, we analyzed the impact of varying video resolution on the COBRA score. We blurred half of the videos (chosen at random), reducing their resolution by a factor of 16 along each axis and then restoring them to their original dimensions. Supplementary Figure 4 shows the results of applying the COBRA score to a dataset containing the blurred and non-blurred videos. Blurring acts as a confounding factor, producing a spurious decrease in model confidence independent of impairment, which reduces the correlation between FMA and the COBRA score. This can be corrected by stratifying the videos, separating them according to whether they are blurred or not.

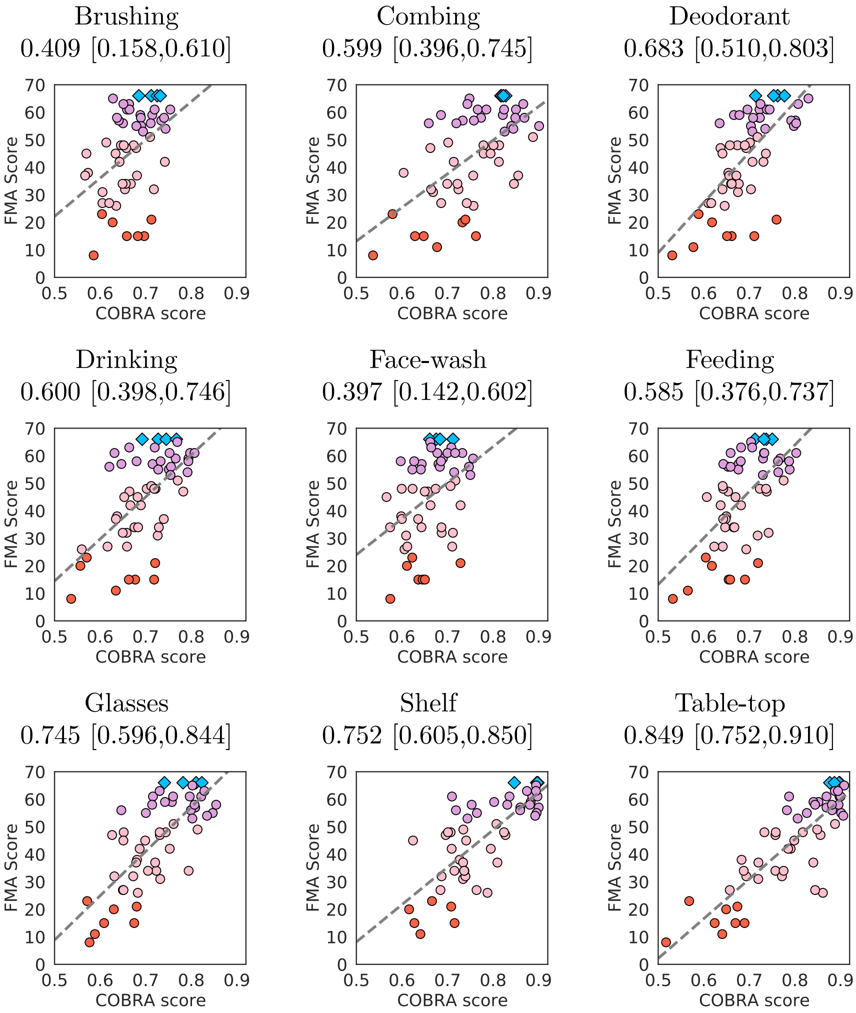

Supplementary Figure 2 Correlation between wearable-sensor COBRA score and clinical assessment for individual rehabilitation activities. Scatterplots of the Fugl-Meyer assessment (FMA) score, based on in-person examination by an expert, and the proposed data-driven COBRA score computed from wearable-sensor data for individual rehabilitation activities. The correlation coefficient ρ is highest for simpler more structured activities such as Glasses, Shelf and Table-top.

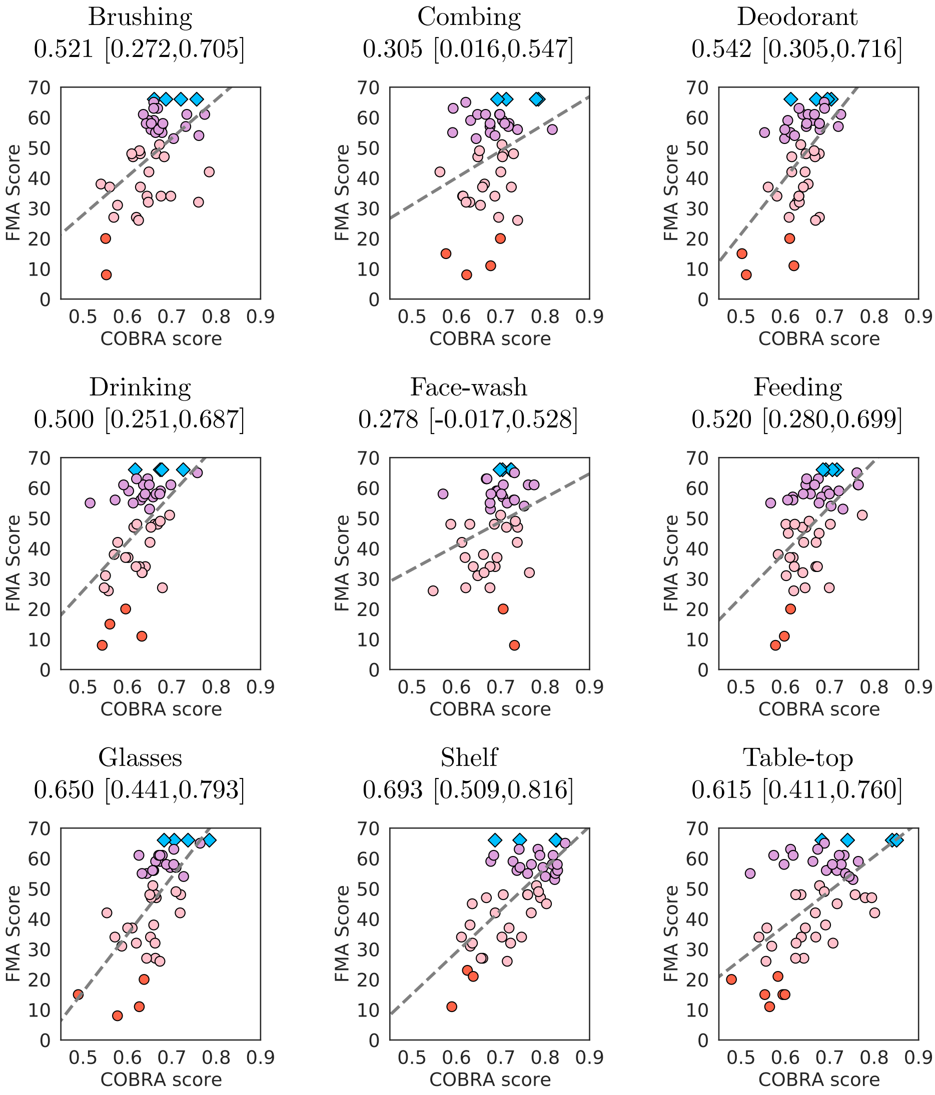

Supplementary Figure 3 Correlation between video COBRA score and clinical assessment for individual rehabilitation activities. Scatterplots of the Fugl-Meyer assessment (FMA) score, based on in-person examination by an expert, and the proposed data-driven COBRA score computed from video data for individual rehabilitation activities. The correlation coefficient ρ is highest for simpler more structured activities such as Glasses, Shelf and Table-top.

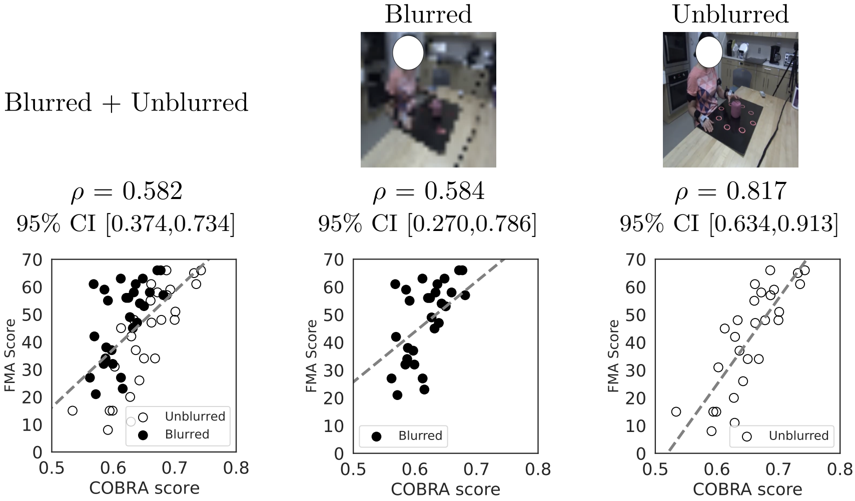

Supplementary Figure 4 Video quality as a confounding factor for the video-based COBRA score. AI models may be sensitive to changes in video quality, such as blurring. The bottom left scatterplot shows the COBRA score computed from blurred and non-blurred videos, as well as the corresponding Fugl-Meyer assessment (FMA) score. Blurring decreases model confidence, systematically decreasing the COBRA score. The bottom middle and right scatterplots show that stratifying the videos according to their quality corrects for the confounding factor, improving the correlation coefficient ρ between the COBRA and FMA scores.

3 Supplementary Methods

3.1 Robustness of the COBRA Score to the Underlying AI Model

In order to evaluate the robustness of the proposed approach to the choice of underlying AI model, we performed experiments with alternative models for both of our applications of interest. In both cases, we found that the COBRA score based on the alternative models is still correlated with the gold-standard reference scores, indicating that the proposed approach is indeed robust to the choice of the underlying AI model.

For quantification of stroke-induced impairment, Supplementary Figure 5 shows a scatterplot of the gold-standard Fugl-Meyer assessment (FMA) score, and the proposed COBRA score computed from wearable-sensor data using a sequence-to-sequence model based on recurrent neural networks (see below for a detailed description), which is completely different from the MS-TCN segmentation model used to obtain our main results. The correlation coefficient between the resulting COBRA score and the FMA score is again high: 0.774 (95% CI [0.636, 0.865]).

For quantification of knee-osteoarthritis severity, Supplementary Figure 6 shows a scatterplot and density plots of COBRA scores computed using a 3D U-Net, described in detail below, which is again different from the Multi-Planar U-Net model used to obtain our main results. The magnitude of the correlation coefficient between the resulting COBRA score and the gold-standard Kellgren-Lawrence grade is lower, but still statistically significant: -0.429 (95% CI [-0.503,-0.349]).

3.1.1 Stroke-Related Motor Impairment

As an alternative AI model to compute the COBRA score for quantification of stroke-induced impairment, we utilize the sequence-to-sequence model proposed in Kaku et al (2022); Parnandi et al (2022). The model consists of an encoder and a decoder, both implemented using recurrent neural networks. The encoder module is a three-layer bidirectional gated-recurrent-unit (GRU) network, with a 1024-dimensional hidden representation, whereas the decoder is another one-layer bidirectional GRU, with a 2048 dimensional hidden representation.

The model was trained on the healthy cohort minimizing a label-smoothed cross-entropy loss (with a smoothing factor of 0.1) via stochastic gradient descent. We used the Adam optimizer with a learning rate of $5 \cdot 10^{-4}$, and adjusted the learning rate with a 1cycle policy Smith and Topin (2019). Additional hyperparameters include a dropout rate of 0.1 and weight decay of 0.0001 (selected via cross-validation).

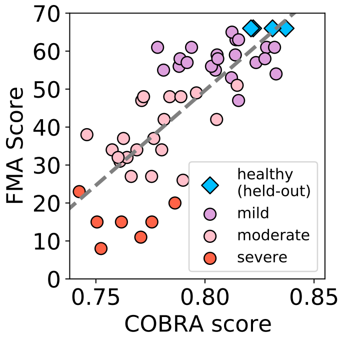

Supplementary Figure 5 Robustness to the choice of AI model for quantification of stroke impairment. Scatterplot of the Fugl-Meyer assessment (FMA) score, based on in-person examination by an expert, and the proposed data-driven COBRA score computed from wearable-sensor data using a different AI model (described in Supplementary Method Section 3.1.1) from the one in main article Figure 3(a)). The correlation between the COBRA and FMA scores is again high, indicating that the proposed approach is robust to the choice of underlying AI model.

3.1.2 Knee-Osteoarthritis Severity

As an alternative AI model to compute the COBRA score for quantification of knee-osteoarthritis severity, we use a 3D U-Net Çiçek et al (2016). This model is a popular baseline for 3D volumetric segmentation tasks in medical applications Perslev et al (2019). The 3D U-Net has an encoder-decoder architecture with skip connections between corresponding layers of the encoder and decoder. Following Perslev et al (2019) we use three layers in the encoder and decoder. We train the 3D U-Net on the training cohort using the same training loss, optimizer, and early stopping rule as for the model described in the Methods section.

3.2 Distance-based Anomaly Quantification

In this section we present an alternative method for anomaly detection and quantification that utilizes an AI model trained only on healthy patients. The method is based on the Fréchet Inception Distance (FID) Heusel et al (2017) to quantify the deviation between a subject and a healthy population. FID is a metric designed to evaluate the similarity between two sets of feature representations extracted by a deep neural network. It has been applied to image generation Heusel et al (2017), where the goal is to determine whether generated images are close to real images or not.

We propose to leverage FID to compare a potentially impaired subject to a healthy reference population using the same model features as in the COBRA framework. First, the data associated with all individuals is fed into a deep neural network, trained to perform a task relevant to the impairment or disease of interest. Then, the features extracted by the neural network are compared via FID to determine to what extent the subject deviates from the population.

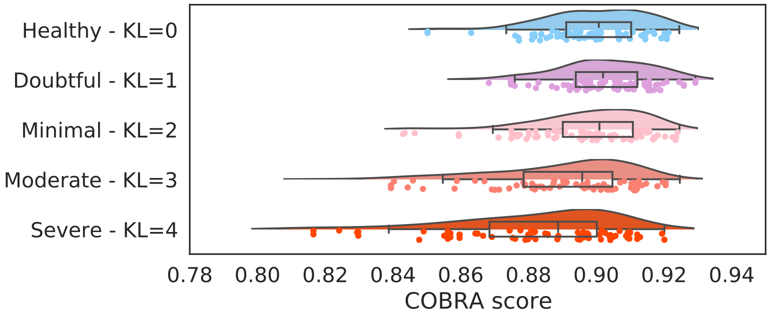

Supplementary Figure 6 Robustness to the choice of AI model for quantification of knee-osteoarthritis severity. Scatterplot and density plots of COBRA scores computed from magnetic-resonance imaging (MRI) knee scans of patients with different Kellgren-Lawrence (KL) grades. The COBRA scores are computed using a different AI model (described in Supplementary Method Section 3.1.2) from the one in Figure 3(b)). The correlation between the COBRA scores and KL grades is again significant, indicating that the proposed approach is robust to the choice of underlying AI model.

Let $x_1, x_2, ..., x_n$ be the features associated with the healthy reference subjects, and $y_1, y_2, ..., y_m$ the feature representations of the potentially-impaired subject. The sample mean and covariance matrices of these features are denoted by μ_x, μ_y and Σ_x, Σ_y, respectively. The FID between the healthy population and the impaired subject is

$$\mathrm{FID}(x, y) = \|\mu_x - \mu_y\|_2^2 + \mathrm{Trace}(\Sigma_x + \Sigma_y - 2(\Sigma_x \Sigma_y)^{1/2}), \tag{1}$$

where $\mathrm{Trace}(\cdot)$ denotes the trace operator and $\|\cdot\|_2$ is the ℓ_2 norm. The lower the FID between the two sets of features, the more similar they are.

As a proof of concept, we apply FID to quantification of stroke-induced impairment using the sensor dataset described in the Results section. The features used to compute the FID are extracted from the penultimate layer of the same model used to compute the COBRA score (the model is described in the Methods section). Two held-out healthy subjects from the test cohort were randomly chosen to be the reference healthy population. The FID of the remaining subjects of the test cohort was computed with respect to this population.

Supplementary Table 8 shows the correlation coefficient of the FID with respect to the reference healthy subjects and the Fugl-Meyer assessment (FMA) for different rehabilitation activities. The magnitude of the correlation is higher when motion primitives are utilized to compute the FID, and for more structured activities. The metrics are not as correlated as the COBRA score and FMA (see Supplementary Figure 2), but these results suggest that there may be multiple ways of exploiting features extracted from AI models to perform anomaly detection and quantification.

Supplementary Table 8 Correlation between Fréchet Inception Distance (FID) and clinical assessment. Correlation coefficient between the FID (with respect to a reference healthy population) computed from wearable-sensor data and the Fugl-Meyer assessment for different rehabilitation activities. The metrics are more correlated when motion primitives are utilized to compute the FID, and for more structured activities. 95% CIs are shown in brackets.

Activity	Non-motion	Motion	All primitives
All	-0.359 [-0.461,-0.025]	-0.715 [-0.780,-0.647]	-0.650 [-0.719,-0.577]
Brushing	-0.041 [-0.249,0.225]	-0.176 [-0.426,0.001]	-0.139 [-0.287,0.078]
Combing	-0.329 [-0.434,-0.140]	-0.427 [-0.703,-0.025]	-0.313 [-0.469,-0.087]
Deodorant	-0.280 [-0.500,-0.081]	-0.693 [-0.793,-0.402]	-0.617 [-0.712,-0.493]
Drinking	-0.243 [-0.433,-0.032]	-0.352 [-0.477,-0.183]	-0.436 [-0.563,-0.277]
Face-wash	-0.246 [-0.433,-0.004]	-0.255 [-0.464,0.025]	-0.347 [-0.491,-0.217]
Feeding	-0.429 [-0.625,-0.230]	-0.396 [-0.561,-0.160]	-0.523 [-0.675,-0.329]
Glasses	-0.510 [-0.619,-0.270]	-0.688 [-0.760,-0.599]	-0.718 [-0.795,-0.624]
Shelf	-0.254 [-0.461,-0.034]	-0.684 [-0.760,-0.580]	-0.597 [-0.699,-0.439]
Table-top	-0.533 [-0.640,-0.418]	-0.693 [-0.758,-0.605]	-0.609 [-0.694,-0.492]

4 Supplementary Notes

4.1 Inference Time

An important consideration for the application of the proposed methodology in a clinical setting is the inference time of the deep-learning models used to compute the COBRA score. In our experiments, we utilized NVIDIA Tesla V100 GPUs. The COBRA score for stroke-induced impairment took 2 seconds to compute per subject for the wearable-sensor data, and 44 seconds for the video data. The COBRA score for knee osteoarthritis took 5 seconds per subject.

References

Çiçek Ö, Abdulkadir A, Lienkamp SS, et al (2016) 3d u-net: learning dense volumetric segmentation from sparse annotation

Heusel M, Ramsauer H, Unterthiner T, et al (2017) GANs trained by a two time-scale update rule converge to a local nash equilibrium. Advances in neural information processing systems 30

Kaku A, Liu K, Parnandi A, et al (2022) StrokeRehab: A benchmark dataset for sub-second action identification. Advances in Neural Information Processing Systems 35:1671–1684

Parnandi A, Kaku A, Venkatesan A, et al (2022) Primseq: A deep learning-based pipeline to quantitate rehabilitation training. PLOS digital health 1(6):e0000,044

Perslev M, Dam EB, Pai A, et al (2019) One network to segment them all: A general, lightweight system for accurate 3d medical image segmentation

Smith LN, Topin N (2019) Super-convergence: Very fast training of neural networks using large learning rates
